# A comparison of seed germination coefficients using functional regression

**DOI:** 10.1002/aps3.11366

**Published:** 2020-07-19

**Authors:** Renáta Talská, Jitka Machalová, Petr Smýkal, Karel Hron

**Affiliations:** ^1^ Department of Mathematical Analysis and Applications of Mathematics Palacký University Faculty of Science 17 Listopadu 12 Olomouc 771 46 Czech Republic; ^2^ Department of Botany Palacký University Faculty of Science Šlechtitelů 27 Olomouc 783 71 Czech Republic

**Keywords:** continuous germination index, functional regression, germination curve, nondecreasing positive smoothing splines, seed germination

## Abstract

**Premise:**

Seed germination over time is characterized by a sigmoid curve, called a germination curve, in which the percentage (or absolute number) of seeds that have completed germination is plotted against time. A number of individual coefficients have been developed to characterize this germination curve. However, as germination is considered to be a qualitative developmental response of an individual seed that occurs at one time point, but individual seeds within a given treatment respond at different time points, it has proven difficult to develop a single index that satisfactorily incorporates both percentage and rate. The aim of this paper is to develop a new coefficient, the *continuous germination index* (CGI), which quantifies seed germination as a continuous process, and to compare the CGI with other commonly used indexes.

**Methods:**

To create the new index, the germination curves were smoothed using nondecreasing splines and the CGI was derived as the area under the resulting spline. For the comparison of the CGI with other common indexes, a regression model with functional response was developed.

**Results:**

Using both an experimentally obtained wild pea (*Pisum sativum* subsp. *elatius*) seed data set and a hypothetical data set, we showed that the CGI is able to characterize the germination process better than most other indices. The CGI captures the local behavior of the germination curves particularly well.

**Discussion:**

The CGI can be used advantageously for the characterization of the germination process. Moreover, *B*‐spline coefficients extracted by its construction can be employed for the further statistical processing of germination curves using functional data analysis methods.

The timing of seed germination is one of the key steps in the plant life cycle, determining when plant growth begins in natural or agricultural ecosystems. In nature, many seeds exhibit various types and levels of dormancy (Baskin and Baskin, 2014). In contrast, crop seeds often germinate as soon as water is imbibed, usually at planting time.

Germination is defined as the events that commence with the uptake of water by the quiescent dry seed and terminate with the elongation of the embryonic axis and the penetration of the radicle. The term germination is often used to indicate its completion; for example, “50% germination” indicates that 50% of a seed population has completed germination (Bewley et al., 2013). In contrast, seed dormancy blocks the completion of germination in an intact viable seed under favorable conditions (Weitbrecht et al., 2011; Baskin and Baskin, 2014). For germination to occur, quiescent seeds must be hydrated at suitable temperatures and in the presence of oxygen. In some cases, specific germination triggers such as fire are required (reviewed by Baskin and Baskin, 2014).

Under natural conditions, seasonal changes play a major role in the plant life cycle. Temperature is a good indicator of the time of year (Burghardt et al., 2015), and thus often regulates seed dormancy and germination (Probert, 2000; Fenner and Thompson, 2005; Baskin and Baskin, 2014). Two distinct effects of temperature have been identified in these processes: first, temperature influences the dormancy per se, and second, temperature determines the germination speed of nondormant seeds (Bradford, 2002). Another environmental factor is water availability, as the water potential of both the soil and seeds varies with the seasons (Bradford, 2002).

Seed populations tend to germinate in a characteristic pattern over time, resulting in a sigmoid curve when the percentage (or absolute number) of seeds that have completed germination is plotted against time. These germination time courses can be used to elucidate the timing, uniformity, and extent of germination in seed populations. The uniformity of germination is indicated by the time between two germination percentiles, such as the period between the points when 10% and 90% of seeds have germinated, with smaller values indicating a greater uniformity. The nondormant viable seeds complete germination, but the dormant or nonviable seeds fail to do so, resulting in a time course that reaches a final percentage of less than 100%.

Seed germination time courses often exhibit complex patterns (Bewley et al., 2013), and most seed germination experiments present difficulties in interpreting and analyzing results (Kader, 2005; Ranal and Santana, 2006). Germination is a qualitative developmental response of seeds that occurs in time, but individual seeds within a given treatment respond within different time intervals. This results in a situation where the final germination percentage alone is not sufficient for a comparative analysis of data sets, a problem for which various germination measurement techniques and methods have been proposed as solutions (reviewed by Kader, 2005; Ranal and Santana, 2006). Time, rate, homogeneity, and synchrony are important aspects that can be measured, informing the dynamics of the germination process. Because they are relevant for crop establishment, these characteristics are important not only for physiologists, seed technologists, and ecologists, but also for agronomists.

A number of coefficients, discussed by Kader (2005), were developed to characterize the entire course of the seed germination process. The *mean germination time* (MGT) (Orchard, 1977) computes the day of average germination; accordingly, the lower the MGT, the faster a population of seeds reaches germination. The *coefficient of velocity of germination* (CVG) (Jones and Sanders, 1987) gives an indication of the rapidity of germination. Its value increases when the number of germinated seeds increases and the time required for germination decreases. Finally, the *germination index* (GI) (Benech Arnold et al., 1991) is defined as a weighted sum of the daily numbers of germinated seeds. The maximum weight is given to the seeds germinated on the first day, while lesser weights are given to the seeds germinated at later dates, with the lowest weight being assigned to seeds germinated on the last day. The GI therefore emphasizes both the percentage of germination and its speed. A higher GI value denotes a higher percentage and a higher rate of germination.

None of these commonly used indices are able to satisfactorily differentiate between germination patterns and capture all important aspects of the germination process. The same is true for other measures such as the *cumulative percentage*, which is the static expression of germination behavior at a given time point; *LT50*, which is the time taken to reach 50% of the final germination level; *total germination* (TG); and the *coefficient of velocity of germination* (CVG), which is used as a measure of the rate and time‐spread of germination.

The aim of this paper is to compare the above coefficients with a new index that considers seed germination to be a continuous process. The comparison is performed using a functional regression model, which reflects how well the respective coefficients predict the course of germination. Moreover, the approximation of the germination process by splines, which is a necessary step for the construction of the new coefficient, enables the derivation of so‐called *B*‐spline coefficients, which can be used for the further statistical processing of germination curves using methods of functional data analysis (Ramsay and Silverman, 2005). This is not possible with any of the previous indices. In this study, the seed germination data are presented as a discrete version of a potentially continuous process and a non‐decreasing monotone smoothing spline is developed to approximate them for further purpose of the analysis. The new coefficient, called the *continuous germination index* (CGI), is introduced, and a functional regression model is proposed to compare the CGI with the other germination coefficients using simulated and real seed germination data.

## METHODS

Germination data can be considered from two basic perspectives: as truly discrete data and as a discrete version of a potentially continuous process. In the case of the former, the seed germination coefficients listed above are designed to address this assumption, at least indirectly. The latter approach, further developed here, considers seed germination data as the result of discretization of a (theoretically) continuous function, with the assumption that such a function is able to better capture the specific course of the germination process (Brown and Mayer, 1988a, b; El‐Kassaby et al., 2008). When seed germination data are considered as a function, a specific approach for their analysis is needed. Functional data analysis (Ramsay and Silverman, 2005) is aimed at developing such methods for a relevant analysis of data where each observation corresponds to a function. Growing curves represent an important example in functional data analysis, as seen in the seminal book by Ramsay and Silverman (2005, p. 2), which presents an example of growing curves based on the heights of 10 girls measured at 31 ages. The discretization is clearly considered in the *x* direction (i.e., time), whereas heights are continuous by nature. Similarly, seed germination data can also be considered to be a type of growing curve. In this case, however, a reverse process needs to be considered in the *y* direction to obtain the resulting functional data; specifically, as there is always a finite number of seeds to germinate, the numbers of germinated seeds are discrete values. However, with an increasing total number of seeds ( $N^{*}$), the approximation by a continuous variable becomes fully reasonable. As a consequence, seed germination can be considered as a continuous process, represented by a positive non‐decreasing curve. Of course, in practice only (discrete) germination data are available, which must first be smoothed to obtain an impression of the (theoretical) continuous germination curve.

### Splines and the optimal smoothing problem

A mathematically elegant approach to obtain easily interpretable smoothed curves (functions) is to use splines, which are functions defined piecewise by polynomials with a range of possible applications, particularly in computer graphics. Splines can also be used to solve the smoothing problem (De Boor, 1978), i.e., to find a function( f(x)) that minimizes the functional

(1) $$J_{l}\left(f\right)=\left(1-\alpha \right)\int_{t_{1}}^{t_{n}}{\left[f^{\left(l\right)}\left(t\right)\right]}^{2}dt+\alpha \sum_{i=1}^{n}{\left[w_{i}\left(y_{i}-f\left(t_{i}\right)\right)\right]}^{2},$$

where parameter $\alpha \in \left(0,1\right)$ and data $\left(t_{i},y_{i}\right),\;i=1,\;\ldots ,\;n$ are given, and where number *l* $\in \left\{0,1,\;\ldots ,\;k-1\right\}$ stands for derivation and *w_i_* ≥0,i=1,…,n are given weights. We note that this minimization problem is in fact a compromise between staying close to the given data and obtaining a smooth function. It was shown that the minimizer is the natural spline $s_{k}\left(t\right)$, i.e., a spline of odd degree k=2l-1, l≥2 that satisfies the additional constraints

$$s_{k}^{\left(l+j\right)}\left(t_{1}\right)=s_{k}^{\left(l+j\right)}\left(t_{n}\right)=0,\;j=0,\;1,\;\ldots ,\;l-2,$$

having as knots the given data $t_{i}.$ Further details about the approach to identifying the optimal smoothing splines are provided in Appendix 1, along with functionals 2–16.

### Nondecreasing optimal smoothing spline

The resulting smoothing spline can have any possible course, and so can also be negative and possibly decreasing on part(s) of its domain. Such spline functions were used for smoothing seed germination data by Hradilová et al. (2019). Although the resulting functions were mostly reasonable, from the methodological perspective it is better to completely avoid the occurrence of the abovementioned situations. Therefore, in the next step the aim was to find an optimal smoothing spline that is nondecreasing over the whole domain, that is, a spline that minimizes functional (2) and satisfies the condition

$$s_{k}^{\prime }\left(t\right)\;\geq \;0\;\forall t\in \left[a,b\right].$$

Its detailed construction is available in Appendix 1.

### Seed germination coefficient derived from splines

The nondecreasing optimal smoothing spline $s_{k}^{*}\left(t\right)$ is a continuous representation of the seed germination data and can be used to analyze the course of the germination. This function reflects well the specific patterns related to germination of a given accession, including the final germination proportion and germination speed. Its advantage is that it assigns a (predicted) number of germinated seeds to each value from the domain $I=\left[a,\;b\right]$, where *a* and *b* are real numbers (e.g., *a* < *b*). Accordingly, the number of seeds is, in general, a positive real value and corresponds to a continuous approximation of the original discrete values. For interpretation purposes, it is therefore preferred to consider relative numbers of germinated seeds (e.g., proportions of germinated seeds in given time points as used in the sequel), which are not related to any fixed number of planted seeds at the beginning of the experiment. Due to scale invariance of the spline functions (and the respective *B*‐spline coefficients), this does not affect the quality of the resulting smoothing. It is important to note that the absolute values of the resulting function matter, because not all seeds necessarily germinate during the experiment. Accordingly, although the nonnegativity and monotonicity of $s_{k}^{*}\left(t\right)$ might evoke the properties of a probability distribution function, it must not necessarily reach 1 in b, the right endpoint of I. Hence, Hradilová et al. (2019) called the germination function represented by $s_{k}^{*}\left(t\right)$ the *absolute germination distribution function* (AGDF).

Because AGDF characterizes the complete information about the course of germination, it can also be used to derive the coefficient(s) that could sufficiently characterize the germination process. Hradilová et al. (2019) proposed that the area under AGDF, hereafter termed the CGI, should be considered; this is computed simply as

(17) $$\text{CGI}=\int_{a}^{b}s_{k}^{*}\left(t\right)dt$$

using a proper numerical integration rule, e.g., the composed trapezoidal rule. Compared with any similar index derived directly from the original (discrete) seed germination data, the advantage of the CGI is that it is able to capture the germination process continuously within the whole domain and thus also better captures the local behavior of germination. Unlike the germination indices discussed above, the CGI assigns the same weight to the whole domain, but in principle any weighting is also possible.

### Practical implementation of the CGI

In order to implement the CGI, it is necessary to first approximate the (theoretically continuous) germination curve using a nondecreasing optimal smoothing spline, in this context called the AGDF. The concrete procedure, which is described in detail in Appendix 1, also guarantees the nonnegativity of the resulting function. Moreover, a proper choice of the parameter $\alpha \in \left(0,1\right)$ in (1) enables the user to choose whether to stay close to the given data or rather to obtain a smooth function (here α=0.5 represents a good general compromise). The next step is to determine the value of the CGI, which is a single number representing the course of the germination curve. This is achieved by computing the area between the spline function and the x axis. Theoretically, this is performed using the definite integral, but in practice an effective numerical integration procedure is employed instead.

### Functional regression for the comparison of seed germination coefficients

For a comparison of different seed germination coefficients, the functional regression model with functional response and scalar predictors in the standard space $L^{2}\left(I\right)$ was utilized. The motivation for this model is very intuitive. The purpose of the seed germination coefficients is to explain the course of the germination curve. In other words, a function is explained by a single number (i.e., the germination coefficient), which could be considered as a real (scalar) predictor variable in a regression model. The standard least squares method cannot be used directly for the estimation of the regression parameters because it assumes a real response; if the response is instead represented by a function, a specific approach is required. For easy interpretability, it is also desirable to make the model as simple as possible; therefore, a linear trend is preferred.

The so‐called *function‐on‐scalar regression* was introduced by Ramsay and Silverman (2005, Chapter 13) and subsequently used in various contexts (see Talská et al., 2018, for the case of probability density functions). Here, the aim is to predict the AGDF, a theoretical function y(t) represented by the nondecreasing optimal smoothing spline $s_{k}^{*}\left(t\right)$, using any of the germination coefficients introduced above. This spline thus plays the role of the functional response y(t), and each of the germination coefficients plays the role of the scalar covariate x. The results (presented in the next section) show that the better the obtained fit, the better the respective coefficient characterizes the germination process. Using the functional regression model may represent an elegant way to evaluate the coefficients; although it necessarily considers germination as a continuous process, from its construction the model does not prefer a priori any of the coefficients, working on the basis of discrete germination data.

Specifically, a function‐on‐scalar regression model is considered, which relates a functional response y(t) to an independent scalar covariate x and the intercept. Consider an N‐dimensional vector of functional observations y(t), a design matrix X of dimension N×2 (the first column is made of ones) and a two‐dimensional vector of unknown functional regression parameters β(t). Let ε(t) be an N‐dimensional vector of independent identically distributed (functional) random errors with a zero‐mean. The functional linear model for the ith observation $y_{i}$, i=1,…,N, associated with the regressor $x_{i}$, is expressed as

(18) $$y_{i}\left(t\right)=\beta_{0}\left(t\right)+x_{i}\beta_{1}\left(t\right)+\varepsilon_{i}\left(t\right),\;i=1,\;\ldots ,\;N,$$

or, in matrix notation, as $y\left(t\right)=X\beta \left(t\right)+\varepsilon \left(t\right)$. The estimators ${\hat{\beta }}_{0}$ and ${\hat{\beta }}_{1}$ of the regression parameters $\beta_{j},\;\;j=0,\;1$, can be obtained by minimizing the least‐square fitting criterion,

(19) $$\text{SSE}\left(\beta \right)=\int_{I}{\left[y\left(t\right)-X\beta \left(t\right)\right]}^{T}\left[y\left(t\right)-X\beta \left(t\right)\right]dt.$$

The smoothness of the resulting estimates is controlled automatically by the smoothness of the observations using the optimal smoothing splines (Talská et al., 2018).

To assess the goodness‐of‐fit of the model on the observed AGDFs, a pointwise version of the coefficient of determination $R^{2}\left(t\right),\;t\in I$ was computed based on the pointwise comparison between the predicted response and the actual data. Additionally, a global coefficient of determination, denoted by $R_{\mathrm{glob}}^{2}$, was computed as

(20) $$R_{\mathrm{glob}}^{2}=\frac{\sum_{i=1}^{N}\;∥{\hat{y}}_{i}-{\bar{y}∥}_{2}^{2}}{\sum_{i=1}^{N}\;∥y_{i}-{\bar{y}∥}_{2}^{2}},$$

where ${∥\cdot ∥}_{2}$ stands for the $L^{2}$ norm defined for any function g from $L^{2}$ space as ${∥g∥}_{2}={\left(\int_{I}{\left|g\left(t\right)\right|}^{2}dt\right)}^{1/2}$, and ${\hat{y}}_{i}$ is ith fitted response function and $\bar{y}$ is the overall mean function $\left(\bar{y}=\frac{1}{N}\sum_{i=1}^{N}y_{i}\left(t\right),\;t\in I\right)$. The latter measures the total sample variance of the $y_{i}\left(t\right)$ explained by the model in a global sense.

## RESULTS

The proposed methodology for the seed germination analysis based on the germination measure CGI defined in (17) was evaluated using both a real germination data set from wild pea (*Pisum sativum* L. subsp. *elatius* (M. Bieb.) Asch. & Graebn.) seeds, previously analyzed by Hradilová et al. (2019), and using hypothetical data previously presented by Kader (2005). The comparison of the CGI with the other commonly used germination indices was based on the methodology using functional regression. All computations were performed using R (R Core Development Team, 2019) and its packages splines, nnls, and fda. The code is available upon request from the authors.

### Analyzing the germination process using a wild pea seed data set

The data used in this example consisted of 115 wild pea accessions ( N) originating from various geographical locations, spanning from the western Mediterranean, through southeastern Europe to the Middle East. A total of 25 seeds per accession were considered ( $N^{*}$), and the number of germinated seeds was scored daily for 28 days ( n). These observed numbers of germinated seeds per day were converted into relative values by dividing each number by 25.

Because the absolute/relative values of cumulative seed germination are considered to be generated in a continuous process, the next step was to estimate the associated underlying (hypothetical) germination function for each of the pea accessions. For this purpose, we denote the relative cumulative number of germinated seeds of the ith accession by $y_{\mathrm{ij}},\;i=1,\;\ldots ,\;N,\;j=0,\;\ldots ,\;n$ until the day $t_{j}=j,\;j=0,\;\ldots ,\;n$. The collected data $\left(t_{j},\;y_{\mathrm{ij}}\right)$ were subsequently smoothed using a system of smoothing splines with support $I=\left[0,28\right]$, fulfilling positiveness and monotonicity, as described in the above sections (“Splines and the optimal smoothing problem” and “Nondecreasing optimal smoothing spline”). For all 115 observations, the same strategy was followed to set the values of the input parameters for the smoothing procedure. We employed smoothing splines of degree k=5 (and the first derivative in the $L^{2}$ norm, i.e., l=1) with the five equally spaced knots in the days $\Delta \lambda =\left\{0,\;7,\;14,\;21,\;28\right\}$, the vectors of weights $w_{i}$ for all data equal to vectors of ones, and the smoothing parameter λ was set to 0.5. Thus, when minimizing the functional (2) (see Appendix 1), the smoothness requirement of the resulting approximation of the germination function using smoothing splines has the same importance as the requirement for staying close to the observed data $\left(t_{j},\;y_{\mathrm{ij}}\right)$. The resulting spline coefficients (Appendix S1) were then used to obtain the resulting spline functions, expressed as

(21) $$s_{5}^{i*}\left(t\right)=\sum_{\nu =-5}^{3}b_{i,\nu }^{*}B_{\nu }^{6}\left(t\right),\;i=1,\;\ldots ,\;N,\;t\in I=\left[0,28\right].$$

**Figure 1:**
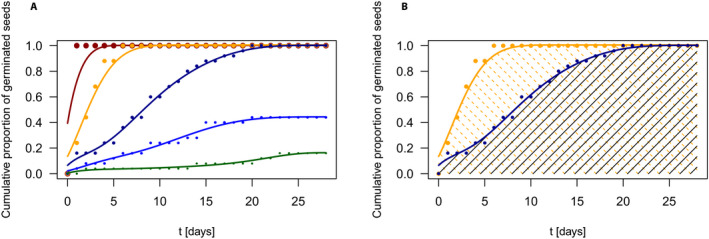
An example of smoothed germination functions for cumulative relative totals. (A) Data smoothed via optimal smoothing splines; each color represents one of the respective germination processes, chosen from a total of 115 to illustrate the variability in the course of processes. (B) The visualization of the germination measure CGI for the germination processes represented by the orange and blue curves. Dots indicate discrete data $\left(t_{j},y_{\mathrm{ij}}\right).$ A total of 25 seeds were included in the germination analysis.

Figure 1A displays an example of the cumulative germination data for a subset of five pea accessions together with the smoothed germination curves. The curves reflect the course of the germination process and the related patterns, such as the proportion of germinated seeds or the germination speed. It must be stressed that whereas the maximum germination proportion is attained for the germination process, represented by red, orange, and dark blue curves for which the underlying data represent proportions of germinated seeds in given time points, the speed of germination differs; the red curve represents the fastest germination process (perhaps the fastest possible process), and the orange and dark blue curves represent gradually slower germination processes. The last two curves, denoted in light blue and green, represent the slower germination processes with the proportion of germinated seeds lower than 50%. This is also well reflected by the germination measure CGI, computed as the area under the germination curve. The CGI values for all 115 accessions are listed in Appendix S1. For the germination processes displayed in Fig. 1A, the following CGI values were calculated: 27.3 (red), 25.6 (orange), 19.5 (dark blue), 8.1 (light blue), and 2.2 (green). Note that, due to the construction of the smoothing spline and the range of values on the *y*‐axis, the CGI cannot exceed the value 28 (see section “Nondecreasing optimal smoothing spline” [above] for more information). A relatively higher value of this germination measure indicates that at least one of the observed parameters, germination proportion or germination speed, is higher than for other accessions. See also Fig. 1B, which illustrates a comparison of the germination measure CGI between the germination processes given by the orange and dark blue curves.

Finally, in order to make a comparison of CGI with other germination measures such as the LT50, TG, MGT, CVG, and GI, we used a functional regression model (18). In particular, the dependency of estimated germination curves $y_{i}\left(t\right),\;i=1,\;\ldots ,\;N$ on the aforementioned germination measures $x_{i}^{m},\;m=1,...,\;M,\;i=1,\;...,\;N$ was modeled by building a total of M=8 functional regression models of the following formula

(22) $$y_{i}\left(t\right)=\beta_{0}^{m}\left(t\right)+x_{i}^{m}\beta_{1}^{m}\left(t\right)+\varepsilon_{i}^{m}\left(t\right),\;i=1,\;\ldots ,\;N,\;m=1,\;\ldots ,\;M,\;t\in I=\left[0,28\right].$$

**Figure 2:**
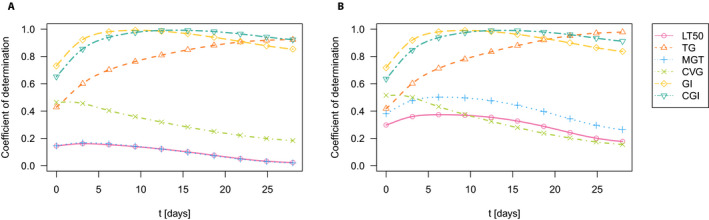
Pointwise coefficient of determination for germination data. (A) All germination data $\left(N=115\right)$. (B) All non‐zero germination data $\left(N=94\right).$ CGI, continuous germination index; CVG, coefficient of velocity of germination; GI, germination index; LT50, time taken to reach 50% of the final germination level; MGT, mean germination time; TG, total germination.

Here, the same *B*‐spline basis system for the response y(t), the regression parameters $\beta_{0}\left(t\right)$ and $\beta_{1}\left(t\right)$, and the error term ε(t) was considered and functional regression models (22) were reduced to multivariate ones where the *B*‐spline coefficients of the estimated germination curves play the role of the (observed) multivariate response variable. Consequently, the estimation of regression parameters $\beta_{0}^{m}\left(t\right)$ and $\beta_{1}^{m}\left(t\right)$ via the SSE criterion (19) reduces to an estimation of the matrix of their corresponding *B*‐spline coefficients (Talská et al., 2018). The output was then used to compute the fitted germination curves ${\hat{y}}_{i}^{m}\left(t\right)$, that is

(23) $${\hat{y}}_{i}^{m}\left(t\right)={\hat{\beta }}_{0}^{m}\left(t\right)+x_{i}^{m}{\hat{\beta }}_{1}^{m}\left(t\right),\;i=1,\;\ldots ,\;N,\;m=1,\;\ldots ,\;M,\;t\in I=\left[0,\;28\right],$$

and subsequently to obtain pointwise and global versions of the coefficient of determination in order to quantify the goodness‐of‐fit of the model on the observed germination curves. Both of them were calculated (i) for all given accessions ( N=115) (Fig. 2A) and (ii) by omitting the 21 accessions with 0% seed germination ( N=94) (Fig. 2B). The graphs of the pointwise coefficient $R^{2}$ suggest that the germination process is best characterized by the CGI together with the TG and the GI. This was further confirmed by the coefficient $R_{\mathrm{glob}}^{2}$ (see Table 1). The CGI reached its highest values among the considered germination measures in both cases, i.e., 95.6% and 95.1%, respectively.

**Table 1:** Global coefficient of determination for all germination data and all non‐zero germination data.

Germination measure	All germination data ( N=115)	All non‐zero germination data ( N=94)
LT50	0.094	0.302
TG	0.824	0.849
MGT	0.094	0.414
CVG	0.292	0.297
GI	0.938	0.933
CGI	0.956	0.951

Note

CGI = continuous germination index; CVG = coefficient of velocity of germination; GI = germination index; LT50 = time taken to reach 50% of the final germination level; MGT = mean germination time; TG = total germination.

### Analyzing the germination process using a hypothetical data set

The hypothetical germination data set published by Kader (2005) consisted of four seed populations with eight different scenarios. Kader subjected these data to a germination analysis in order to evaluate the various germination measures. The design of the scenarios aimed to cover different time spreads of germination as well as the final proportion of germinated seeds. The germination data based on a germination period of 10 days are presented in Table 2. This study shows that the GI best reflects both the final germination proportion of germinated seeds and the germination speed, including the spread, duration, and “high/low” germination events (for more details, see Kader, 2005).

**Table 2:** Hypothetical germination data. The eight scenarios are denoted by the letters A–H, while the four seed populations are listed as sets 1–4. Data are taken from Kader (2005).

Days	A				B				C				D				E				F				G				H			
	Set 1	Set 2	Set 3	Set 4	Set 1	Set 2	Set 3	Set 4	Set 1	Set 2	Set 3	Set 4	Set 1	Set 2	Set 3	Set 4	Set 1	Set 2	Set 3	Set 4	Set 1	Set 2	Set 3	Set 4	Set 1	Set 2	Set 3	Set 4	Set 1	Set 2	Set 3	Set 4
1	0	95	10	0	19	23.7	31.6	47.5	31.6	25	18.3	11.6	10	70	10	15	13.5	19	31.6	95	0	0	0	0	9.5	18.7	27.5	35	0	31.6	0	80
2	0	0	20	0	19	23.7	31.6	47.5	31.6	25	18.3	11.6	15	15	70	70	13.5	19	31.6	0	0	0	0	31.6	9.5	18.7	27.5	0	0	31.6	0	10
3	0	0	65	15	19	23.7	31.6	0	31.6	25	18.3	11.6	70	10	15	10	13.5	19	31.6	0	0	0	0	31.6	9.5	18.7	0	0	0	31.6	0	5
4	15	0	0	35	19	23.7	0	0	0	0	0	0	0	0	0	0	13.5	19	0	0	0	0	31.6	31.6	9.5	18.7	0	0	0	0	0	0
5	80	0	0	45	19	0	0	0	0	0	0	0	0	0	0	0	13.5	19	0	0	0	0	31.6	0	9.5	0	0	0	0	0	0	0
6	0	0	0	0	0	0	0	0	0	0	0	0	0	0	0	0	13.5	0	0	0	0	31.6	31.6	0	9.5	0	0	0	0	0	0	0
7	0	0	0	0	0	0	0	0	0	0	0	0	0	0	0	0	13.5	0	0	0	0	31.6	0	0	9.5	0	0	0	0	0	0	0
8	0	0	0	0	0	0	0	0	0	0	0	0	0	0	0	0	0	0	0	0	31.6	31.6	0	0	9.5	0	0	0	31.6	0	80	0
9	0	0	0	0	0	0	0	0	0	0	0	0	0	0	0	0	0	0	0	0	31.6	0	0	0	9.5	0	0	0	31.6	0	10	0
10	0	0	0	0	0	0	0	0	0	0	0	0	0	0	0	0	0	0	0	0	31.6	0	0	0	9.5	0	0	0	31.6	0	5	0

**Figure 3:**
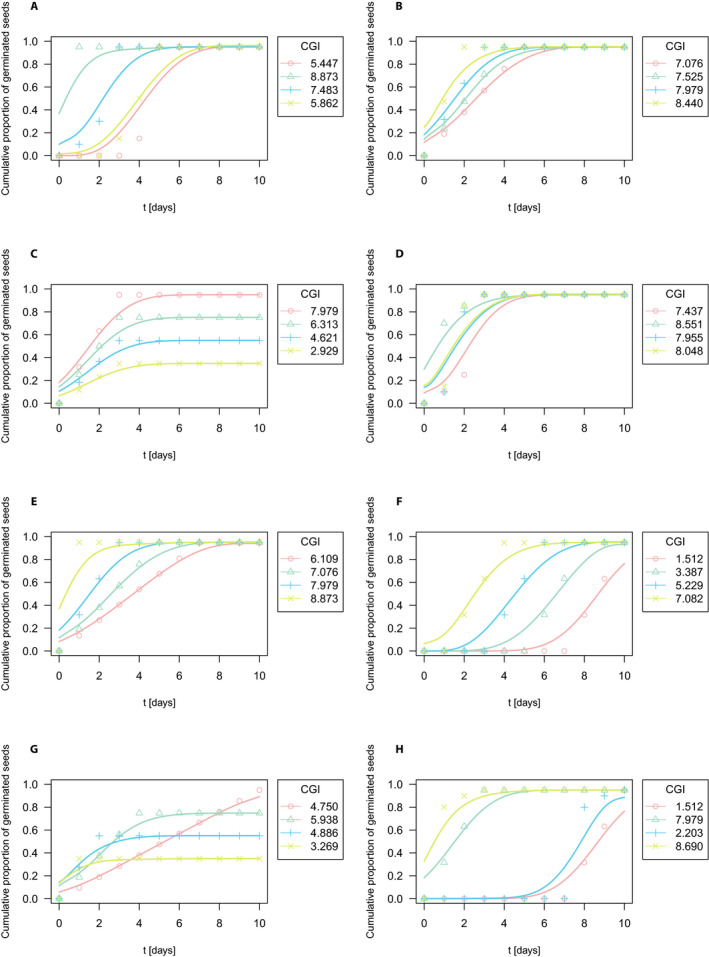
Smoothed germination functions via optimal smoothing splines of hypothetical data using relative cumulative totals together with germination coefficient CGI values computed from the spline approximation. Color resolution corresponds to four seed populations for all eight scenarios A–H: red for set 1, green for set 2, blue for set 3, and yellow for set 4. A total of 100 seeds were included in the germination analysis.

At this point, we will proceed with a calculation of the CGI. To this end, the relative values of cumulative germination were represented by germination curves. Although a small number of time points make the continuous approximation somewhat difficult, it is still within a range acceptable in a functional data analysis context (Ramsay and Silverman, 2005). Here, the smoothing procedure had the same design as the one applied in the case study dealing with wild pea seeds. It is based on quintic splines ( k=5,l=1) on $I=\left[0,10\right]$ with a smoothing parameter α=0.5 and the five knots in the days $\Delta \lambda =\left\{0,\;2,\;5,\;8,\;10\right\}$. The resulting spline approximation of germination curves, together with the raw relative values of cumulative germination, is plotted in Fig. 3 where, in each scenario A–H, the colors distinguish the individual groups of seed populations. The associated CGI is displayed on Fig. 3 and listed in Table 3, together with the same germination measures that were used in the previous wild pea example. As expected due to its nature as a continuous counterpart to the GI, the CGI describes the germination process in a very similar way. For instance, in scenarios B and E, where the four seed populations began germination on the same day and attained the same TG, the higher values of CGI reflect a higher speed of germination. In scenario F, with a uniform distribution of germinated seeds and the same TG, the CGI reflects the time of the first seed germination. A similar situation is represented by scenario H (having the same TG), but in addition to the germination speed, the CGI also accounts for a different distribution of germinated seeds. Table 4 presents a global coefficient of determination based on the functional regression, with missing values indicating a singularity of the respective regression model. Although CGI performed well across the scenarios, it should be noted that the coefficient $R_{\mathrm{glob}}^{2}$ is not of great value in this case due to the lower number of observations ( N=4).

**Table 3:** Germination measures for the hypothetical germination data sets from Table 2.

Germination measure	**A**				**B**				**C**				**D**				**E**				**F**				**G**				**H**			
	Set 1	Set 2	Set 3	Set 4	Set 1	Set 2	Set 3	Set 4	Set 1	Set 2	Set 3	Set 4	Set 1	Set 2	Set 3	Set 4	Set 1	Set 2	Set 3	Set 4	Set 1	Set 2	Set 3	Set 4	Set 1	Set 2	Set 3	Set 4	Set 1	Set 2	Set 3	Set 4
LT50	3	5	1	4	3	3	2	1	2	2	2	2	3	1	2	2	4	3	2	1	9	7	5	3	5	2	1	1	9	2	8	1
TG	0.95	0.95	0.95	0.95	0.95	0.95	0.95	0.95	0.95	0.75	0.55	0.35	0.95	0.95	0.95	0.95	0.95	0.95	0.95	0.95	0.95	0.95	0.95	0.95	0.95	0.75	0.55	0.35	0.95	0.95	0.95	0.95
MGT	4.8	1.0	2.5	4.1	3.0	2.4	1.9	1.5	1.9	1.9	1.9	1.9	2.6	1.3	2.0	1.9	3.9	3.0	1.9	1.0	8.9	6.9	4.9	2.9	5.5	2.4	1.5	1.0	8.9	1.9	8.2	1.2
CVG	20.8	100.0	38.7	23.1	13.3	40.0	50.0	66.6	50.0	50.0	50.0	50.0	31.6	73.0	48.7	51.3	25.0	33.3	50.0	100.0	11.1	14.2	20.0	33.3	18.1	40.0	66.6	100.0	11.1	50.0	12.1	82.6
**GI**	585.0	950	800	595.0	760.0	805.8	853.2	902.5	853.2	675.0	494.1	313.2	795.0	915.0	850.0	860.0	661.5	760.0	853.2	950.0	189.6	379.2	568.8	758.4	495.5	635.8	522.5	350.0	189.6	853.2	265.0	930.0
**CGI**	5.447	8.873	7.483	5.862	7.076	7.525	7.979	8.440	7.979	6.313	4.621	2.929	7.437	8.551	7.955	8.048	6.109	7.076	7.979	8.873	1.512	3.387	5.229	7.082	4.750	5.938	4.886	3.269	1.512	7.979	2.203	8.690

Note

CGI = continuous germination index; CVG = coefficient of velocity of germination; GI = germination index; LT50 = time taken to reach 50% of the final germination level; MGT = mean germination time; TG = total germination.

**Table 4:** Global coefficient of determination for the hypothetical germination data sets from Table 2.

Germination measure	A	B	C	D	E	F	G	H
LT50	0.919	0.891	NA	0.965	0.933	0.899	0.402	0.976
TG	NA	NA	1.000	NA	NA	NA	0.629	NA
MGT	0.945	0.961	NA	0.972	0.933	0.899	0.435	0.979
CVG	0.855	0.933	NA	0.975	0.850	0.813	0.661	0.875
GI	0.948	0.976	1.000	0.972	0.933	0.899	0.664	0.978
CGI	0.952	0.976	1.000	0.971	0.931	0.899	0.683	0.979

Note

CGI = continuous germination index; CVG = coefficient of velocity of germination; GI = germination index; LT50 = time taken to reach 50% of the final germination level; MGT = mean germination time; NA = not available; TG = total germination.

## DISCUSSION

We have introduced a new germination coefficient, CGI, which was developed by considering germination as a continuous process. As such, the CGI represents a continuous counterpart to the more widely used germination coefficient, the GI. The GI thus naturally forms the competitive alternative to CGI, unlike most of other coefficients examined in this study, using both experimental and simulated germination data.

Seed germination is assessed in various biological disciplines in both theoretical and applied fields. Depending on the purpose of the experiments, the type of desired outcome varies. For the seed industry, the final germination percentage is usually a sufficient measure, whereas biological studies require more precise values capturing most aspects of the dynamic germination process (reviewed by Kader, 2005; Ranal and Santana, 2006). Because of these considerations, the use of any given germination data analysis method is prone to misinterpretation if all germination parameters (percentage, speed, and timing) are not taken into account.

Attempts have been made to simplify the characterization of seed germination performance by reducing various germination parameters into a single index (Czabator, 1962); however, reducing multiple germination parameters into one index provides an incomplete picture of germination behavior. Comparisons of the existing commonly used calculation formulae were conducted by Kader (2005), following a previous comparison performed by Brown and Mayer (1988a, b). These studies showed that the GI was the most suitable coefficient for describing the germination percentage and speed relationship because it magnifies the variation among the tested seed lots. Other approaches often use MGT, but this index was shown to be unsuitable for statistical tests (Soltani et al., 2015), which is why the LT50 is often used instead.

Curve‐fitting methods using the Weibull, Gompertz, and probit functions have been used to characterize germination, compare seed pretreatments, and measure seed lot differences (reviewed by Brown and Mayer, 1988a, b, or applied further in the context of generalized linear models by Hay et al., 2014). Similarly, El‐Kassaby et al. (2008) used a four‐parameter Hill function as a curve‐fitting method. Yuan et al. (2016) further conducted genetic mapping of seed dormancy/germination using the Hill function. In those approaches, the germination curve was therefore implicitly considered as the result of a continuous germination process. Despite various assertions, the parameters in these functions do not, however, necessarily lend themselves to simple biological interpretation. Representations of splines with *B*‐spline coefficients, extracted during the calculation of the CGI measure, are simply constructed to best characterize the spline function with respect to a given basis. The purpose of these curve‐fitting models differs from the other coefficients: they do not aim primarily to yield one value that would characterize the course of the germination curve, but rather to provide its interpretable approximation through easily tractable parameterization.

In this study, we showed that the CGI provides better overall results with respect to the quality of the regression fit than the competing coefficients from Kader (2005), including the GI. The CGI is also able to capture the local effects of the germination process more precisely. This further confirms the applicability of the CGI, which was previously used to test wild pea seeds for dormancy using various experimental setups and conditions (Hradilová et al., 2019). Moreover, the spline approximation of the raw germination data used to compute the CGI also enables the derivation of a finite‐dimensional representation of the germination curve using *B*‐spline coefficients. These coefficients can be further used, directly or indirectly, for the statistical processing of a sample of germination curves using functional data analysis methods as an extension of processing single curves using, for example, time‐to‐event (survival) analyses (McNair et al., 2012). Hradilová et al. (2019) accordingly performed a cluster analysis, but other methods can also be considered, such as functional principal component analyses, regression analyses, or any classification method. The CGI thus not only represents a relevant coefficient for the evaluation of the course of the germination process, but also opens new perspectives for germination data analysis. Moreover, the analysis can also be extended to other biological experiments working with growth parameters, which further expands the potential of the approach presented here.

## AUTHOR CONTRIBUTIONS

R.T. performed the analysis and wrote the manuscript; K.H. and J.M. supervised the data processing and edited the manuscript; and P.S. was involved in the study design, funding acquisition, and manuscript writing and editing. All authors contributed to the discussion and preparation of the final manuscript.

## Supporting information


**APPENDIX S1.** *B*‐spline coefficients and germination measures.

## Data Availability

The data used in this study were taken from published work by Hradilová et al. (2019).

## Appendix 1 Approximation of germination curves using splines

### Optimal smoothing splines

Machalová and colleagues (Machalová, 2002a, b; Machalová et al., 2016) studied optimal smoothing splines under different constraints. In a basic setting, finding an optimal smoothing spline means that for given data $\left(t_{i},\;y_{i}\right),$ $a\leq t_{i}\leq b$; weights $w_{i}\geq 0$, i=1,…,n; sequence of knots $\Delta \lambda :=\lambda_{0}=a<\lambda_{1}<\ldots <\lambda_{g}<b=\lambda_{g+1}$, n≥g+1; parameter $\alpha \in \left(0,\;1\right)$; and an arbitrary $l\in \left\{1,\;\ldots ,\;k-1\right\}$, the task is to find a spline $s_{k}\left(t\right)\in S_{k}^{\Delta \lambda }\left[a,b\right]$ that minimizes the functional

(2) $$J_{l}\left(s_{k}\right)=\left(1-\alpha \right)\int_{a}^{b}{\left[s_{k}^{\left(l\right)}\left(t\right)\right]}^{2}dt+\alpha \sum_{i=1}^{n}{\left[w_{i}\left(y_{i}-s_{k}\left(t_{i}\right)\right)\right]}^{2}.$$

The choice of parameter l affects the smoothness of the resulting spline. Symbol $S_{k}^{\Delta \lambda }\left[a,b\right]$ denotes the vector space of polynomial splines of degree k>0, defined on a finite interval $\left[a,b\right]$, used here to time the interval for which the germination is observed, with the sequence of knots Δλ. It is known that $dim\left(S_{k}^{\Delta \lambda }\left[a,\;b\right]\right)=g+k+1$. Every spline $s_{k}\left(t\right)\in S_{k}^{\Delta \lambda }\left[a,\;b\right]$ has a unique representation

(3) $$s_{k}\left(t\right)=\sum_{i=-k}^{g}b_{i}B_{i}^{k+1}\left(t\right),$$

with vector $b={\left(b_{-k},\;\ldots ,\;b_{g}\right)}^{T}$ of the *B*‐spline coefficients. Functions $B_{i}^{k+1}\left(t\right),i=-k,\;\ldots ,\;g$ are *B*‐splines of degree k and form a basis in $S_{k}^{\Delta \lambda }\left[a,b\right]$. Using matrix notation, the spline $s_{k}\left(t\right)$ can be written as

(4) $$s_{k}\left(t\right)=C_{k+1}\left(t\right)b,$$

where $C_{k+1}\left(t\right)=\left(B_{-k}^{k+1}\left(t\right),\;\ldots ,\;B_{g}^{k+1}\left(t\right)\right)$ is the so‐called collocation matrix. If we denote $t={\left(t_{1},\;\ldots ,\;t_{n}\right)}^{T}$, $y={\left(y_{1},\;\ldots ,\;y_{n}\right)}^{T}$, $w={\left(w_{1},\;\ldots ,\;w_{n}\right)}^{T}$, and $W=diag\left(w\right)$, the functional $J_{l}\left(s_{k}\right)$ can be written in a matrix form as

(5) $$J_{l}\left(b\right)=\left(1-\alpha \right)b^{T}N_{\mathrm{kl}}b+\alpha {\left[y-C_{k+1}\left(t\right)b\right]}^{T}W\left[y-C_{k+1}\left(t\right)b\right],$$

where

(6) $$C_{k+1}\left(t\right)=\left(\begin{array}{ccc} B_{-k}^{k+1}\left(t_{1}\right) & \cdots & B_{g}^{k+1}\left(t_{1}\right)\\ \vdots & \ddots & \vdots \\ B_{-k}^{k+1}\left(t_{n}\right) & \cdots & B_{g}^{k+1}\left(t_{n}\right) \end{array}\right)\in R^{n,g+k+1},$$

matrix $N_{\mathrm{kl}}=S_{l}^{T}M_{\mathrm{kl}}S_{l}$ is positive semidefinite with

$$M_{\mathrm{kl}}=\left(\begin{array}{ccc} \left(B_{-k+l}^{k+1-l},\;B_{-k+l}^{k+1-l}\right) & \cdots & \left(B_{g}^{k+1-l},\;B_{-k+l}^{k+1-l}\right)\\ \vdots & \ddots & \vdots \\ \left(B_{-k+l}^{k+1-l},\;B_{g}^{k+1-l}\right) & \cdots & \left(B_{g}^{k+1-l},\;B_{g}^{k+1-l}\right) \end{array}\right)\in R^{g+k+1-l,g+k+1-l}$$

and

$$\left(B_{i}^{k+1-l},\;B_{j}^{k+1-l}\right)=\int_{a}^{b}B_{i}^{k+1-l}\left(t\right)B_{j}^{k+1-l}\left(t\right)dt$$

stands for the scalar product of *B*‐splines in $L^{2}\left(\left[a,b\right]\right)$ space. The matrix $M_{\mathrm{kl}}$ is positive definite, because $B_{i}^{k+1-l}\left(t\right)\geq 0$, i=-k+l,…,g are basis functions. Furthermore, matrix $S_{l}=D_{l}L_{l}\ldots D_{1}L_{1}\in R^{g+k+1-l,g+k+1}$ is an upper triangular matrix that has full row rank. $D_{j}\in R^{g+k+1-j,g+k+1-j}$ is a diagonal matrix, such that

(7) $$D_{j}=\left(k+1-j\right)diag\left(d_{-k+j,}\;\ldots ,\;d_{g}\right)$$

with

$$d_{i}=\frac{1}{\lambda_{i+k+1-j}-\lambda_{i}}\forall i=-k+j,\ldots ,g$$

and

(8) $$L_{j}:=\left(\begin{array}{cccc} -1 & 1 & 0 & 0\\ 0 & \ddots & \ddots & 0\\ 0 & 0 & -1 & 1 \end{array}\right)\in R^{g+k+1-j,\;g+k+2-j}.$$

### Construction of the nondecreasing optimal smoothing spline

Because the derivative of the spline can be expressed as a spline of a lower degree (De Boor, 1978; Dierckx, 1993), we can easily find sufficient conditions for the nondecreasing spline. In matrix notation, we have

$$s_{k}^{\prime }\left(t\right)=C_{k}\left(t\right)D_{1}L_{1}b,$$

where $C_{k}\left(t\right)=\left(B_{-k}^{k}\left(t\right),\;\ldots ,\;B_{g}^{k}\left(t\right)\right)$. According to the positivity of the *B*‐splines, $B_{i}^{k}\left(t\right)\geq 0$ (De Boor, 1978), formulae (7) and (8), it follows that if $L_{1}b\geq 0$, $s_{k}\left(t\right)$ is a nondecreasing spline. Finally, the above considerations imply that a nondecreasing optimal smoothing spline is a spline whose vector of the *B*‐spline coefficients b minimizes function $J_{l}\left(b\right)$, (5), and satisfies the condition $L_{1}b\geq 0$. The function $J_{l}\left(b\right)$ is in fact a quadratic function, which can be written as

$$J_{l}\left(b\right)=b^{T}\mathrm{Hb}-2\alpha b^{T}C_{k+1}^{T}\left(t\right)\mathrm{Wy}+y^{T}\mathrm{Wy}.$$

The matrix

$$H\;:=\left(1-\alpha \right)N_{\mathrm{kl}}+\alpha C_{k+1}^{T}\left(t\right){\mathrm{WC}}_{k+1}\left(t\right)$$

is regular and positive definite; therefore, there exists an upper triangular matrix T such that $T^{T}T=H$. The last term in $J_{l}\left(b\right)$ is constant, so it can be omitted for minimization. This leads to a problem of quadratic programming with the linear inequality constraints

(9) $$\min b^{T}T^{T}\mathrm{Tb}-2\alpha b^{T}C_{k+1}^{T}\left(t\right)\mathrm{Wy}$$

(10) $$L_{1}b\geq 0.$$

Let

$$z\;:=\alpha {\left(T^{T}\right)}^{-1}C_{k+1}^{T}\left(t\right)\mathrm{Wy},$$

then

$${∥\mathrm{Tb}-z∥}_{2}^{2}=b^{T}T^{T}\mathrm{Tb}-2\alpha b^{T}C_{k+1}^{T}\left(t\right)\mathrm{Wy}+z^{T}z,$$

where ${∥a∥}_{2}^{2}=a^{T}a$. The last term is constant, so it does not affect the minimization process. By denoting

u:=Tb-z

the so‐called least distance programming problem (Lawson and Hanson, 1995) is obtained in the form of

(11) $$\min{∥u∥}_{2}^{2}$$

(12) Gu≥h,

where

$$G\;:=L_{1}T^{-1},\;h\;:=-L_{1}T^{-1}z$$

because from the definition of u we have

$$u+z=\mathrm{Tb}\Rightarrow b=T^{-1}\left(u+z\right)\Rightarrow L_{1}b=L_{1}T^{-1}u+L_{1}T^{-1}z.$$

Hence, it is clear that

$$L_{1}b\geq 0\Leftrightarrow L_{1}T^{-1}u\geq -L_{1}T^{-1}z.$$

Accordingly, if $u^{*}$ is the solution of the least distance programming problem (11), (12), then

(13) $$b^{*}=T^{-1}\left(u^{*}+z\right)$$

is the solution of problem (9), (10). Consequently, the resulting nondecreasing optimal smoothing spline is obtained by the formula

(14) $$s_{k}^{*}\left(t\right)=\sum_{i=-k}^{g}b_{i}^{*}B_{i}^{k+1}\left(t\right),$$

where the vector $b^{*}={\left(b_{-k}^{*},\;\ldots ,\;b_{g}^{*}\right)}^{T}$ is given in (13).

In order to obtain a positive nondecreasing optimal smoothing spline, a further condition

$$s_{k}\left(t\right)\geq 0$$

needs to be added. Due to the nonnegativity of *B*‐splines, it is known that if b≥0 then spline $s_{k}\left(t\right)=C_{k+1}\left(t\right)b$ is nonnegative. Hence, a condition b≥0 is added to the optimization problem (9), (10) as an additional constraint. Finally, the optimization problem is given as

(15) $$\min b^{T}T^{T}\mathrm{Tb}-2\alpha b^{T}C_{k+1}^{T}\left(t\right)\mathrm{Wy}$$

(16) Ab≥0

with matrix

$$A=\left(\begin{array}{c} L_{1}\\ I \end{array}\right),\;I\in R^{g+k+1}.$$

The next steps are the same as for a nondecreasing optimal smoothing spline with the only difference that

$$G\;:={\mathrm{AT}}^{-1},\;h\;:=-{\mathrm{AT}}^{-1}z.$$
